# Cognitive impairment after tooth extraction: appraising literature and recommendations for future research

**DOI:** 10.2340/aos.v83.41393

**Published:** 2024-09-09

**Authors:** Mahmoud T. Hefnawy, Mahmoud Elfil, Abdulqadir J. Nashwan, Mohamed Elfil

**Affiliations:** a Faculty of Medicine, Zagazig University, Zagazig, Egypt; b Faculty of Dentistry, Pharos University, Alexandria, Egypt; c Nursing & Midwifery Research Department (NMRD), Hamad Medical Corporation, Doha, Qatar; d Department of Public Health, College of Health Sciences, QU Health, Qatar University, Doha, Qatar; e Department of Neurology, University of Miami/Jackson Health System, Miami, FL, USA

Dear Editor,

Cognitive impairment leads to several daily limitations, caregiver stress, and financial strain, imposing a significant global burden on both individuals and society. The recent advancement of healthcare systems and life expectancy necessitate the importance of directing clinical research toward preventing cognitive decline via identifying and protecting, if possible, against potential etiologies. The primary etiology of cognitive impairment is attributed to age-related white matter disease. Additionally, neurodegenerative conditions such as Alzheimer’s disease, and various other neurodegenerative disorders contribute significantly to the global burden of cognitive decline.

Recently, preclinical studies have reported tooth loss or extraction as a potential underlying factor for cognitive impairment. The applicability of these preclinical studies on humans has shown similar findings. The underlying pathological explanation of cognitive decline after tooth loss is unknown. However, human and animal studies theorize that occlusal support or masticatory mechanisms influence cognitive performance [1]. Chewing plays a mechanical role in the blood flow dynamics to brain areas that are responsible for cognitive functions. Besides, the hippocampus and chewing organs are linked through several neuronal circuits responsible for learning and memory processes [1, 2]. Thus, losing natural teeth leads to impairment in chewing and occlusal support, which might ultimately result in cognitive impairment.

Furthermore, tooth extraction and local anesthesia are significant stressors that can lead to a state of chronic stress and inflammation, which, in the long term, can lead to neurodegenerative changes. Also, periodontitis-related tooth loss may introduce new pathogens, such as Klebsiella, to intestinal flora, which disrupts the gut-brain axis [2], the disruption of which might contribute to cognitive decline through immune-mediated mechanisms [3].

Investigating the potential impact of these pathophysiological mechanisms among young adults is important for two main reasons: Firstly, elderly groups are already at risk of cognitive decline secondary to age-related neurodegeneration, which confounds the ability of researchers and clinical investigators to assess the impact of tooth loss in those groups. Secondly, preclinical studies showed cognitive decline among juvenile rats after tooth loss, which makes it important to investigate if similar findings are present among young adults [2, 4].

We have screened primary clinical studies investigating the role of tooth loss on cognitive decline among humans by searching four databases: PubMed, Scopus, Web of Science, and Cochrane Library using a well-constructed search strategy including cognitive decline and tooth loss terms (Supplementary file 1).

Our final screening yielded 35 studies, of which 22 (62.9%) were cohort, 10 (28.6%) were cross-sectional, and three (8.6%) were case-control studies. Notably, we could not find studies that specifically considered young adults. The baseline ages of the population before follow-up were +60 years in 26 (74.3%) studies and +50 years in six (17.1%) studies, while only three (8.6%) studies included people less than 50 years old at baseline (Figure 1; Supplementary Table 1). The most common cognitive assessment tool was the Mini-Mental State Examination (MMSE) in 16 (45.7%) of studies, followed by the Diagnostic and Statistical Manual of Mental Disorders (DSM) revised 3^rd^ and 4^th^ editions in four (11.4%) of studies (Figure 2; Supplementary Table 1).

**Figure 1:**
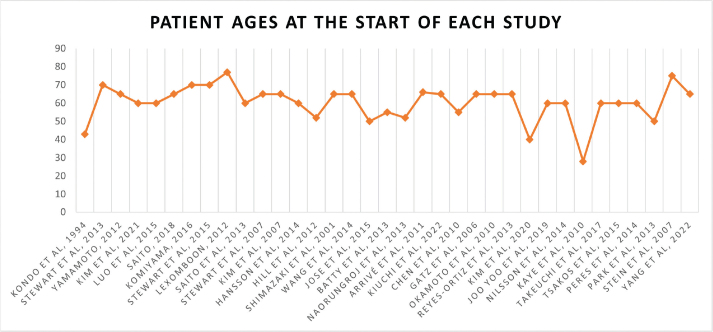
Graph showing baseline ages of included population.

**Figure 2:**
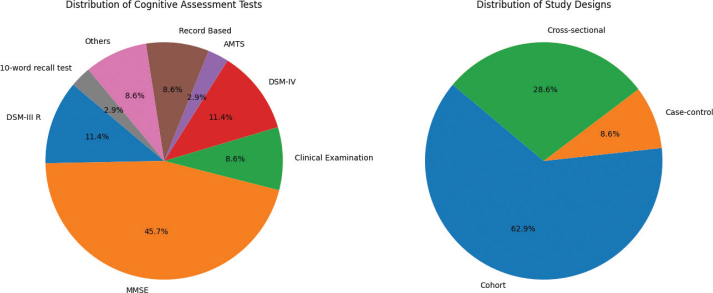
Pie charts showing cognitive assessment tools and study designs.

The cutoff age for defining the elderly state varies among studies. However, 60 years or above is the generally defined age at which populations are considered elderly. As previously mentioned, investigating the relationship between tooth loss and cognitive decline in this category may overlap with age as a confounding factor, as age is a strong risk for cognitive decline. In a recent meta-analysis by Li et al. [5], a regression analysis was conducted to limit the potential effect of age as a confounding factor, yet it is essential to note that regression analysis will not completely neutralize the age bias, and the confounding effects may persist even after adjustment, especially in unbalanced and non-randomized samples as in observational studies [6].

Most of the included studies used MMSE as a screening tool for cognitive decline. However, it is important to note that MMSE measures global cognitive function. Therefore, we further recommend considering specific cognitive tests for particular cognitive domains at risk like learning and memory. The degree of cognitive impairment should be assessed before and after tooth loss to determine the exact severity level.

Since the key management option for cognitive impairment is early prevention if possible, understanding the potential risks in all age groups is crucial. The main message of this study is to consider young adults in long-term prospective studies to understand the relationship between cognitive decline and tooth loss among young-age groups.

## Supplementary Material

Cognitive impairment after tooth extraction: appraising literature and recommendations for future research

## Data Availability

All the steps of obtaining data in this study are provided in supplementary file 1. Any additional information regarding the study can be found by contacting the corresponding author.
