# Supplementary material for: Cognitive impairment after tooth extraction: appraising literature and recommendations for future research
Source: Acta Odontol Scand. 2024 Sep 9;83:41393. doi: 10.2340/aos.v83.41393 (PMC11409816; doi:10.2340/aos.v83.41393)
Supplement: Cognitive impairment after tooth extraction: appraising literature and recommendations for future research [file AOS-83-41393-s1.pdf]

Supplementary material has been published as submitted. It has not been copyedited or typeset by Acta Odontologica Scandinavica.

Supplementary Materials

1. Flow chart of the screening process:

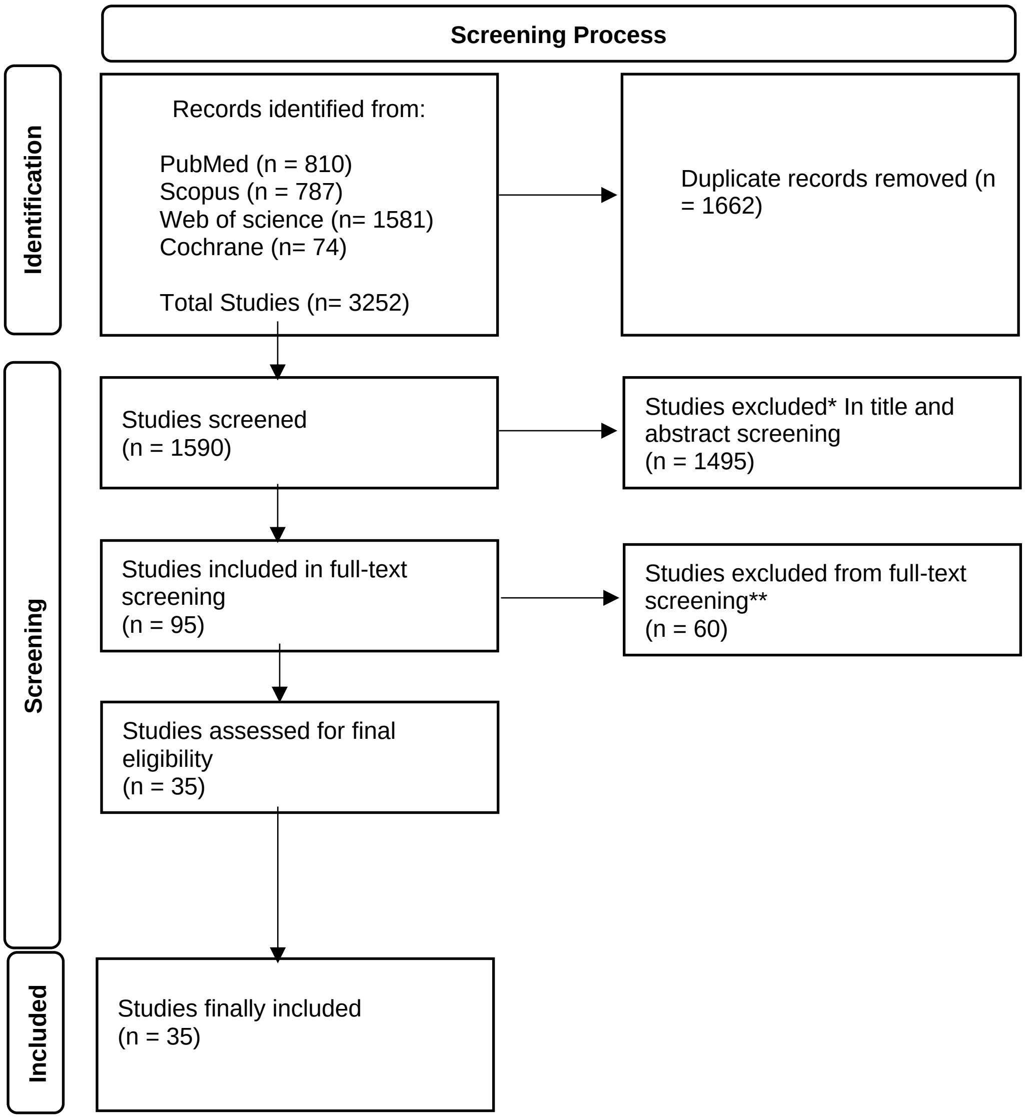

*: Exclusion criteria were animal studies, reviews, reports, conference abstracts, and non-English studies with no available translation.
**: Irrelevant studies to our research question.

2. Supplementary Table 1: Baseline Data from included studies

Title	Author, year	Baseline Age	Cognitive Assessment Methods	Study Design	N
A case-control study of Alzheimer's disease in Japan-	Kondo et al,	43	DSM-III R	Case-	60

significance of life-styles.	1994			control	
Adverse oral health and cognitive decline: the health, aging and body composition study	Stewart et al, 2013	70	MMSE	Cohort	1053
Association between self-reported dental health status and onset of dementia: a 4-year prospective cohort study of older Japanese adults from the Aichi Gerontological evaluation study (AGES) project	Yamamoto, 2012	65	Validated Dementia Questionnaire	Cohort	4425
Association between tooth loss and Alzheimer's disease in a nested case-control study based on a national health screening cohort	Kim et al, 2021	60	Clinical Examination	Case-control	39,810
Association between tooth loss and cognitive function among 3063 Chinese older adults: a community-based study.	Luo et al, 2015	60	DSM-IV	Cross-sectional	3,063
Association between tooth loss and cognitive impairment in community-dwelling older Japanese adults: a 4-year prospective cohort study from the Ohasama study	Saito, 2018	65	MMSE	Cohort	140
Association between tooth loss, receipt of dental care, and functional disability in an elderly Japanese population: the Tsurugaya project	Komiyama, 2016	70	MMSE	Cohort	834
Associations between oral health and risk of dementia in a 37-year follow-up study: The prospective population study of women in Gothenburg.	Stewart et al, 2015	70	DSM-III R	Cohort	1462
Chewing ability and tooth loss: association with cognitive impairment in an elderly population study.	Lexomboon, 2012	77	MMSE	Cross-sectional	557
Cognitive function and number of teeth in a community-dwelling population in Japan.	Saito et al, 2013	60	MMSE	Cross-sectional	462
Dental health and cognitive impairment in an English national survey population.	Stewart et al, 2007	65	AMTS	Cross-sectional	2463
Dental health, nutritional status and recent-onset dementia in a Korean community population	Kim et al, 2007	65	MMSE	Cross-sectional	686
Dental status is unrelated to risk of dementia: a 20-year prospective study	Hansson et al, 2014	60	DSM-IV	Cohort	2,120
Dentition, dental health habits, and dementia: the leisure world cohort study.	Hill et al, 2012	52	MMSE	Cohort	5468
Influence of dentition status on physical disability, mental impairment, and mortality in institutionalized elderly people.	Shimazaki et al, 2001	65	Diagnosis based on medical records	Cohort	2220
Investigating tooth loss and associated factors among older Taiwanese adults. Arch Gerontol	Wang et al, 2014	65	MMSE	Cross-sectional	2286
Is periodontitis a risk factor for cognitive impairment and dementia? a case-control study.	Jose et al, 2015	50	DSM-IV	Case-control	409
Oral disease in relation to future risk of dementia and cognitive decline: prospective cohort study based on the action in diabetes and vascular disease: Preterax and Diamicron modified-release controlled evaluation (ADVANCE) trial	Batty et al, 2013	55	MMSE	Cohort	11,140
Oral health and cognitive function in the Atherosclerosis Risk in Communities (ARIC) study	Naorungroj et al, 2013	52	DWR & DSS	Cohort	9,874
Oral health condition of French elderly and risk of dementia: a longitudinal cohort study	Arrivé et al, 2011	66	DSM-III R	Cohort	405
Oral status and dementia onset: mediation of nutritional and social factors.	Kiuchi et al, 2022	65	Dementia Scale	Cohort	35,744
Patterns of tooth loss in older adults with and without dementia: a retrospective study based on a Minnesota cohort	Chen et al, 2010	55	Diagnosis based on medical records	Cohort	491
Potentially modifiable risk factors for dementia in identical twins.	Gatz et al, 2006	65	Clinical Examination	Cohort	3373
Relationship of tooth loss to mild memory impairment and cognitive impairment-findings from the Fujiwara-Kyo study.	Okamoto et al, 2010	65	MMSE	Cross-sectional	4031
Self-reported tooth loss and cognitive function: data from the Hispanic Established Populations for Epidemiologic Studies of the Elderly (Hispanic EPESE)	Reyes-Ortiz et al, 2013	65	MMSE	Cohort	3,050

Severe periodontitis with tooth loss as a modifiable risk factor for the development of Alzheimer, vascular, and mixed dementia: national health insurance service-national health screening retrospective cohort 2002–2015	Kim et al, 2020	40	Clinical Examination	Cohort	20230
The effect of missing teeth on dementia in older people: a nationwide population-based cohort study in South Korea	Joo Yoo et al, 2019	60	Diagnosis based on medical records	Cohort	209,806
Tooth loss and cognitive functions among older adults.	Nilsson et al, 2014	60	MMSE	Cross-sectional	1147
Tooth loss and periodontal disease predict poor cognitive function in older men	Kaye et al, 2010	28	MMSE	Cohort	597
Tooth loss and risk of dementia in the community: the Hisayama study	Takeuchi et al, 2017	60	DSM-III R	Cohort	1,566
Tooth loss associated with physical and cognitive decline in older adults	Tsakos et al, 2015	60	10-word recall test	Cohort	3,166
Tooth loss is associated with severe cognitive impairment among older people: findings from a population-based study in Brazil.	Peres et al, 2014	60	MMSE	Cross-sectional	1705
Tooth loss may predict poor cognitive function in community-dwelling adults without dementia or stroke: the PRESENT project.	Park et al, 2013	50	MMSE	Cross-sectional	438
Tooth loss, dementia and neuropathology in the Nun study.	Stein et al, 2007	75	MMSE	Cohort	144
Tooth loss, denture use, and cognitive impairment in Chinese older adults: a community cohort study	Yang et al, 2022	65	MMSE	Cohort	17 79

DSM-III R (Diagnostic and Statistical Manual of Mental Disorders, Third Edition, Revised); MMSE (Mini-Mental State Examination); Validated Dementia Questionnaire; DSM-IV (Diagnostic and Statistical Manual of Mental Disorders, Fourth Edition); AMTS (Abbreviated Mental Test Score); Diagnosis based on medical records; DWR & DSS (Delayed Word Recall & Delayed Story Recall)

3. Supplementary Table 2: Showing Search Strategy & Results in the four databases:

**Data Base**	**Search Strategy**	**Number of citations**
PubMed	((Tooth loss) OR (Dental extraction) OR (Tooth extraction) OR (Dental loss)) AND ((Cognitive decline) OR (Cognitive impairment) OR (Cognitive loss) OR (Dementia))	810
Web Of Science	((Tooth loss) OR (Dental extraction) OR (Tooth extraction) OR (Dental loss)) AND ((Cognitive decline) OR (Cognitive impairment) OR (Cognitive loss) OR (Dementia))	1581
Scopus	((Tooth loss) OR (Dental extraction) OR (Tooth extraction) OR (Dental loss)) AND ((Cognitive decline) OR	787

	(Cognitive impairment) OR (Cognitive loss) OR (Dementia))	
Cochrane	((Tooth loss) OR (Dental extraction) OR (Tooth extraction) OR (Dental loss)) AND ((Cognitive decline) OR (Cognitive impairment) OR (Cognitive loss) OR (Dementia))	74
